# Minimally invasive quantification of cerebral P2X7R occupancy using dynamic [^18^F]JNJ-64413739 PET and MRA-driven image derived input function

**DOI:** 10.1038/s41598-021-95715-y

**Published:** 2021-08-09

**Authors:** Nathalie Mertens, Mark E. Schmidt, Anja Hijzen, Donatienne Van Weehaeghe, Paulien Ravenstijn, Marleen Depre, Jan de Hoon, Koen Van Laere, Michel Koole

**Affiliations:** 1 Nuclear Medicine and Molecular Imaging, Department of Imaging and Pathology, University Hospital and KU Leuven, Herestraat 49, 3000 Leuven, Belgium; 2 Janssen Research and Development, Beerse, Belgium; 3 Center for Clinical Pharmacology, University Hospital and KU Leuven, Leuven, Belgium

**Keywords:** Molecular medicine, Diagnostic markers, Biomedical engineering

## Abstract

[^18^F]JNJ-64413739 has been evaluated as PET-ligand for in vivo quantification of purinergic receptor subtype 7 receptor (P2X7R) using Logan graphical analysis with a metabolite-corrected arterial plasma input function. In the context of a P2X7R PET dose occupancy study, we evaluated a minimally invasive approach by limiting arterial sampling to baseline conditions. Meanwhile, post dose distribution volumes (V_T_) under blocking conditions were estimated by combining baseline blood to plasma ratios and metabolite fractions with an MR angiography driven image derived input function (IDIF). Regional postdose V_T,IDIF_ values were compared with corresponding V_T,AIF_ estimates using a arterial input function (AIF), in terms of absolute values, test–retest reliability and receptor occupancy. Compared to an invasive AIF approach, postdose V_T,IDIF_ values and corresponding receptor occupancies showed only limited bias (Bland–Altman analysis: 0.06 ± 0.27 and 3.1% ± 6.4%) while demonstrating a high correlation (Spearman ρ = 0.78 and ρ = 0.98 respectively). In terms of test–retest reliability, regional intraclass correlation coefficients were 0.98 ± 0.02 for V_T,IDIF_ compared to 0.97 ± 0.01 for V_T,AIF._ These results confirmed that a postdose IDIF, guided by MR angiography and using baseline blood and metabolite data, can be considered for accurate [^18^F]JNJ-64413739 PET quantification in a repeated PET study design, thus avoiding multiple invasive arterial sampling and increasing dosing flexibility.

## Introduction

The purinergic receptor subtype 7 receptor (P2X7R) is a membrane bound, adenosine triphosphate (ATP)-gated ion channel embedded in the cell membrane and mainly expressed on activated microglia and astrocytes in the central nervous system (CNS). P2X7R plays a role in the release of the pro-inflammatory cytokine interleukin (IL)-1β and IL-18 through inflammasome activation^1,2^. Since P2X7R is expressed in virtually all immune cell types, it is a potential therapeutic target for the treatment of neuroinflammatory diseases. Different preclinical studies in animal models of Alzheimer’s disease, Parkinson’s disease, multiple sclerosis, Huntington’s disease, stroke and mood disorders, have indicated a potential therapeutic use of CNS-penetrating P2X7R antagonists^3–9^. Therefore, a specific positron emission tomography (PET) ligand could be used to provide further insight into the underlying pathophysiology of these disorders, gain evidence of target engagement of candidate P2X7 antagonists, and assist in clinical dose selection. In the past, several PET ligands targeting P2X7R, e.g., [^11^C]GSK1482160^10^, [^11^C]JNJ-54173717^11^, [^18^F]JNJ-64413739^12,13^, [^18^F]EFB^14^ and [^18^F]PTTP^15^, were developed and evaluated in a preclinical setting. To our knowledge, only [^18^F]JNJ-64413739^16^, [^11^C]JNJ-54173717^17,18^ and [^11^C]SMW139^19^ have hitherto be used for in vivo quantification of P2X7R expression in the human brain.

In general, both a two-tissue compartmental model and Logan graphical analysis were identified as the most suitable approaches for the in vivo tracer kinetic modeling of PET tracer targeting P2X7R, using a metabolite-corrected arterial plasma input function (AIF) obtained by arterial blood sampling. As the P2X7R is widely expressed throughout the brain, a generic reference tissue approach could not be applied to simplify quantitative PET imaging procedures. Therefore, arterial cannulation and blood sampling remains mandatory for accurate quantification although it hampers clinical applicability due to the invasive nature of the procedure and the additional logistical burden. Moreover, to investigate the role of neuroinflammation during disease progression of specific brain disorders^20,21^ and to support the development of new therapeutic strategies^22^, multiple PET studies with repeated invasive arterial cannulation procedures would be needed which can seriously hinder patient recruitment.

To reduce invasiveness while maintaining high quantitative accuracy, the use of an image derived input function (IDIF) has been widely investigated and suggested as a valid alternative to arterial sampling^23^. However, IDIF approaches without any blood sampling have proven to yield less accurate estimates of the underlying input function as compared to IDIFs calibrated by limited blood sampling. Indeed, a more accurate correction for partial volume effects, as well as a better determination of time dependent fraction of parent tracer in plasma per subject, were obtained using this additional blood information. Therefore, this study aims to develop and validate a less invasive IDIF approach for quantifying P2X7R expression with [^18^F]JNJ-64413739, denoted hereafter [^18^F]JNJ-739, in the context of a longitudinal PET dose occupancy study. This less invasive approach limits arterial blood sampling to baseline conditions and takes full advantage of these baseline blood data to obtain an accurate IDIF for PET quantification under blocking conditions, thus avoiding repetitive arterial cannulation.

Whole blood IDIF was obtained by combining dynamic PET data with magnetic resonance angiography (MRA) data with both datasets acquired simultaneously on an integrated PET-MR system. A partial volume corection (PVC) of the carotid artery signal was optimized by comparing baseline IDIF with sampled AIF to obtain a subject specific correction. This baseline PVC, together with baseline blood and metabolite data, was used to correct the postdose IDIF. Next, non-invasive postdose distributions volumes (V_T,IDIF_) were obtained with the corrected postdose IDIF and compared with the corresponding post dose distribution volumes obtained with an invasive AIF sampling procedure (V_T,AIF_). As second objective, the test–retest variability (TRV) of V_T,IDIF_ was compared to TRV of V_T,AIF_, and receptor occupancies (RO) based on postdose V_T,IDIF_ (RO_IDIF_) were compared with estimates using postdose V_T,AIF_ (RO_AIF_).

## Materials and methods

### Study design and [^18^F]JNJ-739 PET-MR imaging

In total, 11 healthy male subjects (age 32 ± 11 years, range 22–51 years; weight 75 ± 5 kg, range 68–84 kg) underwent a 90-min dynamic [^18^F]JNJ-739 PET-MR scan (GE Healthcare Signa) with arterial blood sampling before and after the oral administration of a selective P2X7R antagonist (5–300-mg), namely JNJ-55308942 (n = 10, NCT03437590) and JNJ-54175446 (n = 1, NCT03088644) (see Table 1 and Supplementary Figure S1 online). Details on the study design, as well as the characteristics, synthesis and radiolabeling of [^18^F]JNJ-739 have been described previously^16^. The study was conducted at the University Hospital Leuven, Belgium and approved by the local Ethics Committee (University Hospitals Leuven/KU Leuven) and each subject signed a written informed consent before enrollment. All procedures performed in studies involving human participants were in accordance with the ethical standards of the institutional and/or national research committee and with the 1964 Helsinki declaration and its later amendments or comparable ethical standards.

**Table 1:** Overview of the study design with corresponding acquired blood and dynamic PET data for this study.

No	Study design	Subject	Scan session	MRA acquisition	Blood sampling (automatic (A); manual (M))
1	Test–retest study	Subject 1	Test	No, use retest MRA	A
2			Retest	Yes	A
3		Subject 2	Test	Yes	A
4			Retest	Yes	A
5		Subject 3	Test	Yes	A
6			Retest	No, use test MRA	M
7		Subject 4	Test	No, use retest MRA	M
8			Retest	Yes	A
9	Dose occupancy study	Subject 5	Baseline	Yes	M
10			Blocking at T1	Yes	M
11			Blocking at T2	Yes	M
12		Subject 6	Baseline	Yes	M
13			Blocking at T1	Yes	M
14			Blocking at T2	Yes	M
15		Subject 7	Baseline	Yes	M
16			Blocking at T1	Yes	M
17			Blocking at T2	Yes	M
18		Subject 8	Baseline	No, use blocking MRA	M
19			Blocking at T1	Yes	M
20			Blocking at T2	Yes	M
21		Subject 9 (excluded)	Baseline	Yes	M
22			Blocking at T1	No, use baseline MRA	M
23			Blocking at T2	Yes	M
24		Subject 10	Baseline	Yes	M
25			Blocking at T1	Yes	M
26		Subject 11	Baseline	Yes	M
27			Blocking at T1	Yes	M

Dynamic PET data were acquired in list mode, rebinned in 38 frames (9 × 10 s, 6 × 15 s, 6 × 30 s, 3 × 1 min, 2 × 3 min, 9 × 5 min, 3 × 10 min) and corrected for deadtime, randoms and scatter. A zero-echo-time (ZTE) MR-based approach was used to generate a MR-based attenuation map and to correct for attenuation^24^. Each frame was reconstructed using ordered subsets expectation maximization (OSEM, 28 subsets, 6 iterations) and included time-of-flight (TOF) information, resolution modeling and an in-plane Gaussian post-smoothing with a full width half maximum (FWHM) of 4 mm. The dynamic PET data were corrected for motion by aligning each frame with the mean of the first 17 frames representing the first 5 min of the PET acquisition using a rigid transformation.

Simultaneous with the PET acquisition, a 3D volumetric T1 weighted BRAVO MR sequence (plane: sagittal; TE: 3.2 ms; TR: 8.5 ms; TI: 450 ms; flip angle: 12; receiver bandwidth: 31.2 kHz; NEX: 1; voxel size: 1 × 1 × 1 mm) was acquired which was used to determine a subject specific tissue probability map for gray matter, white matter and cerebrospinal fluid.

In 22 out of 27 dynamic PET scans, a 3D Time-of-Flight (TOF) MRA sequence was acquired before the start of the dynamic PET acquisition (MRA: Plane: axial; field of view (FOV): 200 × 180 mm; slice thickness: 1.2 mm; voxel size: 0.4 × 0.7 × 0.6 mm; flip angle: 8, receiver bandwidth: 31.2 kHz, acquisition time: 2 m 51 s), while no MRA data was available for the other scans due to practical timing issues. For these dynamic PET scans, the MRA data of another dynamic PET-MR scanning session of the same subject were re-used (see Table 1).

In the context of a receptor occupancy study, seven subjects (range 22–51 years) underwent baseline and postdose scanning with average [^18^F]JNJ-739 activity of 139 ± 15 MBq (range 84–155 MBq). This corresponded to an injected mass dose of 1.9 ± 1.9 μg (range 0.6–9.3 μg) with the range of specific activity of 4–102 GBq/μmol (average: 45 ± 22 GBq/μmol).

Postdose scanning was acquired after oral administration of a selective P2X7R antagonist drug (unpublished data). Five subjects received two postdose scans, while two subjects only received one postdose scan, resulting in 12 pairs of baseline and postdose scans.

During the dynamic PET acquisition, 22 arterial blood samples were manually obtained over a 90-min period post injection (10, 20, 30 40, 50, 60, 70, 80, 90, 100, 110, 120, 150, 180, 240, 300, 600, 1200, 1800, 2400, 3600 and 4800 s) to determine activity levels in whole blood and plasma. In parallel, 6 arterial blood samples obtained over a 90-min period post injection (2, 5, 15, 30, 60 and 80 min) were used to determine the percentage of intact tracer in plasma.

For test–retest repeatability, four subjects (age 22–32 years) received two [^18^F]JNJ-739 PET-MR scans using the same imaging protocol as described above (interscan interval 26–97 days). These subjects received 168 ± 23 MBq (range 116–196 MBq) of [^18^F]JNJ-739 (injected mass dose of 1.2 ± 0.7 μg (range 0.2–2.1 μg) and a specific activity of 106 ± 131 GBq/μmol (range 24–422 GBq/μmol)). During dynamic scanning, continuous arterial blood sampling was performed (Twilite Swisstrace, Zurich, Switzerland). In addition, arterial blood samples were manually collected at discrete time points to determine the radioactivity in whole blood and plasma, as well as for determination of the percentage of intact tracer in plasma.

Detailed information about the blood and PET data processing is given in the Supplementary Materials and Methods online.

### IDIF extraction

**Figure 1:**
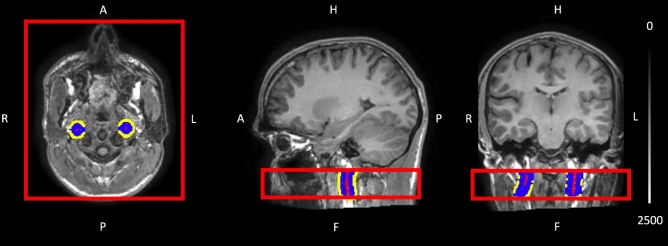
3D T1-weighted MR image with 3D MR angiography overlay (FOV shown in red) in MNI space, showing left and right internal carotid arteries (red), spill-over regions (blue) and background regions (yellow) and used to calculate an image derived input function.

MRA images were co-registered with the PET images by performing a rigid co-registration with the mean of the first 5 PET frames. Internal left and right carotid artery VOIs (Fig. 1) were defined based on an automated segmentation of the MRA images (auto iso-contour, PMOD v4.1) and projected on the dynamic PET data to extract TACs for the internal carotid arteries (${C}_{ca}$).

To model the spill-over from the surrounding tissue, the binary mask of left and right carotid artery was extended by applying an in-plane Gaussian smoothing with a FWHM of 7 mm and a lower threshold of 0.01 to create an enlarged binary mask. Next, this binary mask was dilated by 3 voxels and the original mask was subtracted from the dilated mask to define a background VOI with limited spill-over effects from the carotid arteries. For each pair of MRA scans (baseline/postdose scan, and test/retest scan, respectively), carotid artery and background VOIs were limited to the overlap of both MRA FOVs, determined after a rigid co-registration of both MRA scans. The left and right internal carotid artery VOIs, as well as left and right background VOIs, were then merged for further analysis.

Partial volume effects (PVE) between PET activity concentration in the carotid arteries (${C}_{ca}$) and corresponding background were modeled as:

1 $${C}_{ca}\left(t\right)=w*{C}_{wb}\left(t\right)+(1-w)*{C}_{bg}(t)$$

where ${C}_{wb}$ and ${C}_{bg}$ represented the whole blood activity concentration and the PET activity concentration in the background VOI, respectively, while w was the partial volume weighting factor which models the spill-in and spill-over effects ($0\le w\le 1$) between background and carotid arterial activity^25–27^.

If the optimal w is known, the whole blood activity concentration, free of PVE, can be determined as:

2 $${C}_{wb}\left(t\right)=\frac{{C}_{ca}\left(t\right)}{w}-\frac{{C}_{bg}\left(t\right)}{w}+{C}_{bg}(t)$$

With ${C}_{ca}$ and ${C}_{bg}$ the activity concentrations of the internal carotid artery and background VOI, respectively as measured with the dynamic PET scan. To determine the optimal $w$, we used baseline AIF data as estimates for ${C}_{wb}\left(t\right)$ of Eq. (1) while ${C}_{ca}$ and ${C}_{bg}(t)$ were estimated by applying the appropriate carotid and background VOI to the baseline dynamic PET data. The optimal $w$ was determined by minimizing the following cost function corresponding to the sum of squared differences (Python 3.8):

3 $$\underset{0\le \mathrm{w}\le 1}{\mathrm{min}}\sum_{T}{\left({C}_{ca}\left(t\right)-w*{C}_{wb}\left(t\right)-\left(1-w\right)*{C}_{bg}\left(t\right)\right)}^{2}$$

As such, the optimal weighting factor was obtained for correcting the baseline or test IDIF for spill-over and spill-in effects of surrounding tissue and was then used to correct the postdose or retest IDIF.

### PET quantification and statistical data analysis

Regional V_T_ values were determined by using Logan graphical analysis^28^ (t* = 48.5 min) and a metabolite-corrected arterial plasma input function (Python 3.8). Detailed information about the PET quantification is given in the Supplementary Materials and Methods online.

To evaluate the impact of using baseline metabolite and blood data for blocking conditions, we compared postdose V_T_ values obtained using the postdose AIF and postdose blood and metabolite data (V_T,AIF_) with postdose V_T_ estimates based on a postdose AIF but combined with baseline blood and metabolite data (V_T,bAIF_).

Finally, postdose V_T,IDIF_ were estimated by combining baseline blood and metabolite data with IDIF extracted from the postdose dynamic PET and compared with postdose V_T,AIF_ estimates. An overview of the different approaches is given in Table 2.

**Table 2:** An overview of the different approaches used to calculate distribution volumes.

Acronyms	
AIF	Arterial input function representing the time dependent activity concentration in arterial whole blood, obtained from arterial blood sampling
IDIF	Image derived input function representing time dependent activity concentration in arterial whole blood obtained from dynamic PET and MR angiography
V_T,AIF_	Distribution volume obtained from dynamic PET and the corresponding AIF and blood/metabolite data
V_T,bAIF_	Distribution volume obtained from dynamic PET and the corresponding AIF but using baseline blood/metabolite data
V_T,IDIF_	Distribution volume obtained from dynamic PET and the corresponding IDIF but using baseline blood/metabolite data

In terms of repeatability, test V_T,AIF_ and retest V_T,IDIF_ with the latter using a retest IDIF combined with blood and metabolite data of the test scan, were compared to evaluate both reliability and test–retest variability by calculating the intraclass correlation coefficient (ICC) and the (absolute) test–retest variability ((a)TRV).

In terms of RO, estimates representing the global, drug-induced target occupancy for the whole brain were obtained by a axes-transformed Lassen plot^29^ while assuming a constant V_ND_ for baseline and dosing conditions. Baseline V_T,AIF_ and postdose V_T,IDIF_ were used to estimate the dose dependent receptor occupancy (RO_IDIF_) and compared to a standard approach where both baseline and postdose V_T,AIF_ are used to determine receptor occupancy (RO_AIF_).

Results were presented as average ± standard deviation with the coefficient of variation (CV%) to describe variability. A two-way repeated measure analysis of variance (ANOVA) was used to evaluate the interaction between brain regions and different methods to determine the input function as well as the main effect of using different input functions. In addition, multiple comparisons were performed with regional V_T,AIF_ as reference values. A Bonferroni correction was used for comparing baseline regional V_T,IDIF_ and V_T,AIF_ values while a Dunnett correction for postdose regional V_T,IDIF_, V_T,bAIF_ and V_T,AIF_ values. Next, RO_IDIF_ and RO_AIF_ estimates were compared using a Wilcoxon signed-rank test as data did not approximate a normal distribution. Additionally, the Spearman correlation coefficient ρ was calculated to evaluate the relationship between different methods to estimate cortical V_T_ and RO while a Bland–Altman analysis was used to evaluate the agreement between these methods. All statistical analyses were performed using Prism v8 (GraphPad) and using a significance level of p < 0.05.

## Results

### IDIF extraction

**Figure 2:**
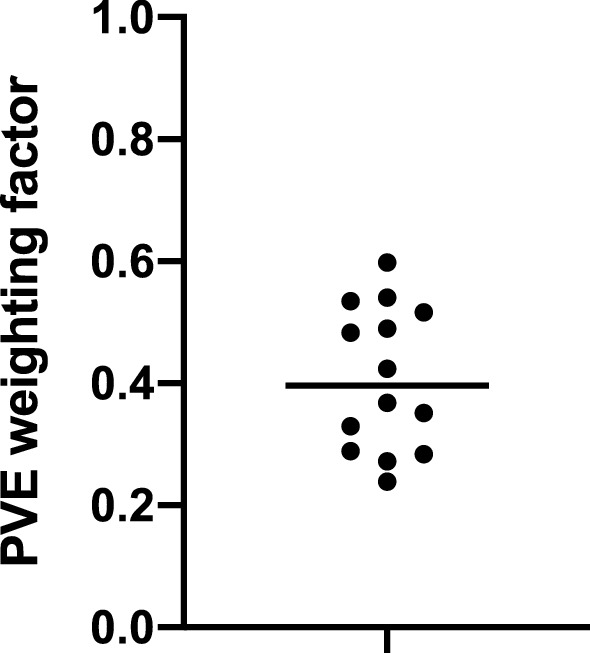
Subject specific partial volume effect (PVE) weighting factors showing a rather high between-subject variability. Weighting factors are obtained by optimizing baseline (n = 10) and test (n = 4) image derived input function (IDIF) with corresponding sampled arterial input function (AIF), and used to correct postdose and retest whole blood IDIF.

One data set was excluded from the analysis since there was no overlap between the FOV of baseline and postdose MRA because two different sets of axial planes were selected as FOV for the two MRA scans. For all other baseline (n = 10) and test (n = 4) scans, the weighting factors, representing the fraction of the carotid PET signal coming from arterial blood, were in the range of [0.24,0.60] (0.41 ± 0.12, Fig. 2). These weighting factors were used to correct the corresponding postdose and retest IDIF such that a whole blood arterial input function was obtained.

### Baseline IDIF validation

**Figure 3:**
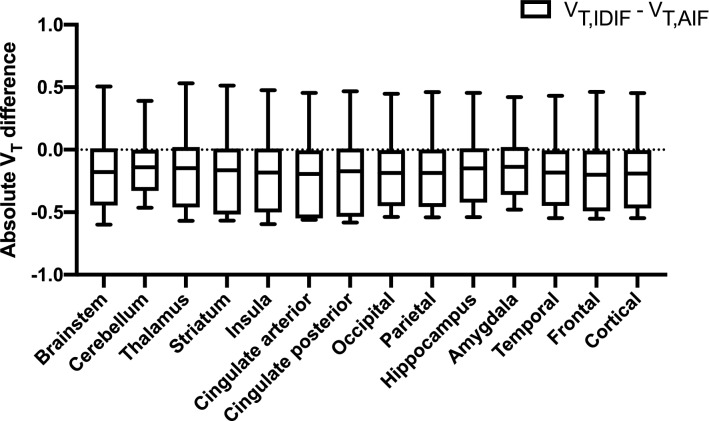
Absolute differences between regional V_T_ values for pooled baseline and test scans (n = 14) calculated with a Logan graphical analysis and using either a sampled arterial input function (V_T,AIF_) or an image derived input function (V_T,IDIF_). Both input functions used arterial blood samples to obtain the arterial metabolite-corrected plasma input function.

When comparing regional, baseline V_T,IDIF_ and V_T,AIF_ (Fig. 3), a two-way repeated measure ANOVA showed no significant interaction between brain regions and the different approaches to estimate baseline V_T_ (p > 0.99), while a significant effect of the use of different input functions (p < 0.05) and different brain regions (p < 0.05) was observed. However, a Bonferroni posttest showed no significant differences between baseline V_T,IDIF_ and V_T,AIF_ values for all brain regions. These findings were supported by a Bland–Altman analysis, indicating a limited bias of − 0.07 ± 0.36 for the cortical region, with similar CV% for baseline cortical V_T,AIF_ and V_T,IDIF_ (12.9% and 13.2%, respectively). Additionally, a strong significant correlation was found between baseline V_T,AIF_ and V_T,IDIF_ (ρ = 0.83, p = 0.0004).

### Postdose PET quantification

**Figure 4:**
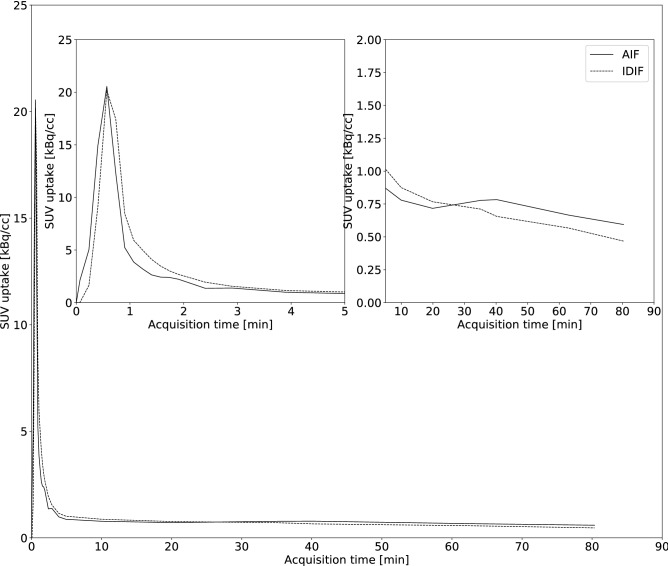
Average postdose parent plasma input function as a function of time of all subjects (n = 10) showing similar shape for the image derived input function (IDIF) and the arterial input function (AIF).

**Figure 5:**
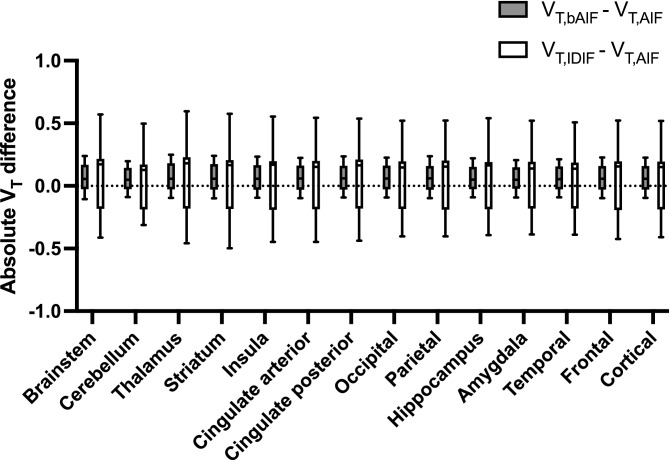
Absolute differences between regional V_T_ values for postdose scans (n = 10) estimated with a Logan graphical analysis. Postdose V_T_ values were obtained using either an arterial metabolite corrected plasma input function based on postdose arterial blood samples (V_T,AIF_), an arterial metabolite corrected plasma input function based on baseline arterial blood samples (V_T,bAIF_) and an image derived input function based on baseline arterial blood samples (V_T,IDIF_).

Figure 4 illustrates the similar shape for the average postdose AIF and IDIF (n = 10) with the IDIF corrected for partial volume effects using baseline data. A two-way repeated measures ANOVA comparing postdose V_T,AIF_, V_T,bAIF_ and V_T,IDIF_ for different brain regions (Fig. 5), showed no interaction between brain regions and the different approaches to estimate postdose V_T_, while a significant effect of the use of different input functions (p < 0.05) was observed. However, a Dunnett posttest found no significant differences between V_T,IDIF_ and V_T,AIF_ for all brain regions. A Bland–Altman analysis showed a positive bias of 0.06 ± 0.11 when comparing cortical V_T,bAIF_ with V_T,AIF_ while in addition, a very strong significant correlation was found between both measures (ρ = 0.99, p < 0.0001). Between cortical V_T,AIF_ and V_T,IDIF_, again a strong significant correlation was found (ρ = 0.78, p = 0.01) with a positive bias of 0.06 ± 0.27 when comparing cortical V_T,IDIF_ with V_T,AIF_ using a Bland–Altman analysis. Additionally, postdose cortical V_T,AIF_ and V_T,IDIF_ showed similar variability (CV% of 21.1% and 19.1%, respectively).

### Test–retest repeatability

In terms of repeatability, V_T,AIF_ and V_T,IDIF_ showed good repeatability for all brain regions with ICC values of 0.97 ± 0.01 and 0.98 ± 0.02, respectively. Evaluation of the test–retest variability resulted in an average aTRV of 7.6% ± 1.6% and an average TRV of 5.7% ± 2.7% for V_T,AIF_ values across brain regions, while for V_T,IDIF_, the average aTVR and TRV across brain regions was 4.9% ± 2.2% and − 4.4% ± 2.7%, respectively.

### P2X7R occupancy estimates

**Figure 6:**
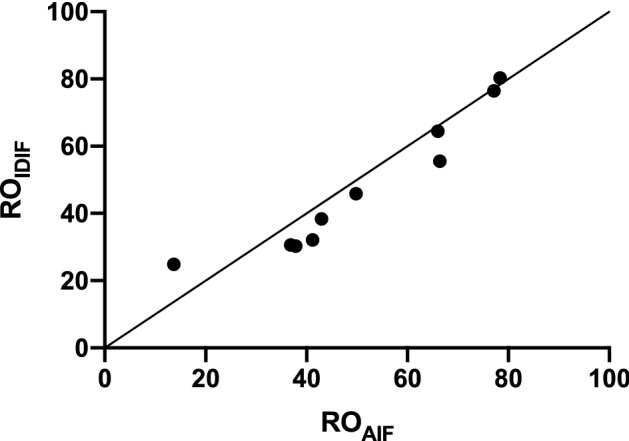
Scatter plot of receptor occupancy (RO) estimates (solid line is line of identity) between RO values calculated based on regional baseline and postdose V_T,AIF_ (RO_AIF_) and RO values calculated based on regional baseline V_T,AIF_ and postdose V_T,IDIF_ (RO_IDIF_). RO estimates are calculated using a Lassen plot.

Figure 6 shows RO_AIF_ and RO_IDIF_ results (n = 10), showing very similar RO estimates for both methods. A Bland–Altman analysis showed a negative bias of − 3.1% ± 6.4% when comparing RO_IDIF_ with RO_AIF_. Additionally, no significance difference was found between RO_IDIF_ and RO_AIF_ estimates (p = 0.16), while a very strong significant correlation was demonstrated between both occupancy measures (ρ = 0.98, p < 0.0001).

## Discussion

In this study, we validated a minimally invasive approach to quantify [^18^F]JNJ-739 PET images in the context of a receptor occupancy and test–retest study. Therefore, we developed a technique to estimate the arterial blood and parent plasma input function based on dynamic PET data and co-registered MRA images, acquired simultaneously with an integrated PET-MR system. The MRA acquisition has an optimal FOV which is situated lower than PET FOV as lower segments of the carotid structure are more reliable for quantification because of the larger diameter of these segments and the lower risk of including other structures with high activity in background region such as brain tissue or the external carotid arteries. Therefore, MRA data needed to be acquired before the start of the PET acquisition since it is not possible to change the FOV during a dynamic PET acquisition on the GE Signa PET-MR.

Since, for each subject, [^18^F]JNJ-739 showed very similar tracer metabolization and plasma characteristics in arterial blood under baseline and blocking conditions (Supplementary Figure S2 online), we assumed one scanning session with arterial blood sampling to minimize repeating arterial blood sampling and used only baseline arterial blood information to quantify both baseline and postdose scans. This less invasive approach will present a clear added value in longitudinal PET studies where neuroinflammation will be monitored during therapy in patients with breast cancer^30^, or during disease progression in patients with amyotrophic lateral sclerosis^17^. Based on the results of this study, we can confirm that this approach is a valid alternative to avoid arterial blood sampling for the postdose PET scan. For our study, the observed between subject variability was 21% and 19% for AIF- and IDIF-based cortical V_T_, respectively, which was in line or smaller than for other IDIF approaches^29,31^, where even higher differences of more than 10% were reported between AIF- and IDIF-based V_T_ values for [^11^C]PIB. In terms of test–retest variability, differences between the IDIF- and AIF-based quantification were in the range of only a few percent which is in line with published test–retest data^32,33^ reporting an increase in test–retest variability of 0–2% for IDIF-based PET quantification. The low test–retest variability and appropriate use of baseline arterial blood data to rescale the postdose IDIF was also confirmed by high agreement between IDIF- and AIF-based receptor occupancy estimates.

Although the current approach still requires one PET scanning session with arterial blood sampling, it offers more flexibility in terms of study design (e.g., multiple dosing points without the need for repetitive arterial cannulation) and makes the overall quantification less dependent of successful arterial sampling during each scanning session. As a fully non-invasive approach would be clearly advantageous from a patient comfort and logistics viewpoint, one could consider introducing population-based values for tracer metabolization and blood to plasma ratios to completely eliminate arterial cannulation. However, since this tracer demonstrated a high inter-subject variability of plasma to blood activity ratios and tracer metabolization rates (Supplementary Figure S3 online), a population-based approach would not be valid. Therefore, an IDIF approach combined with arterial blood samples was essential for this tracer to properly scale the IDIF in a subject specific way^34,35^. For future studies, one could consider a further reduction of arterial blood samples to optimize the IDIF approach^26,36^. However, since this approach still requires arterial cannulation and therefore does not significantly reduce the overall invasiveness of the PET procedure, we choose to take full advantage of the presence of the arterial line to sample the complete AIF to allow an optimized, subject specific PVC and to accurately determine the subject specific, time dependent tracer metabolization rate and plasma to blood ratio.

It has been demonstrated that the PVC of an IDIF approach can also be optimized without any blood sampling by accurately modeling the PET resolution and considering different background subregions to account for varying background activity while taking advantage of MRA data to accurately segment the internal carotid arteries^32,37^. Since arterial blood data were available and because of the 25 cm FOV of the integrated PET-MR system, we considered a lower portion of the carotid structure such that we could simplify this approach and reduce the background region to one region, while assuming uniform background activity. Furthermore, we took advantage of the available arterial blood samples from the baseline PET session to accurately estimate the contribution of background activity to the IDIF signal. This finding is in line with Hackett et al.^26^, showing higher agreement between AIF and IDIF for patients with head and neck tumors by introducing arterial blood activity information in the IDIF model.

Since the geometry of the carotid structure determines the PVE of the corresponding PET signal, we assumed the PVE of carotid PET signal to be reproducible within one subject between different scanning sessions. This way, we could optimize the PVC for the IDIF extraction using the arterial blood data of one scanning session and translate this PVC to estimate the whole blood activity concentration for the other sessions without the need for repeated arterial blood sampling. To ensure a similar geometry of the carotid structure between scanning sessions, we limited the extraction of the PET signal from the internal carotid arteries and background artery to overlapping MRA FOVs between sessions. To maximize the overlap of both MRA acquisitions, one should carefully localize both MRA FOVs such that the same set of axial planes is covered.

For our study, we observed that the weighting factor, representing the fraction of carotid PET signal corresponding to the activity concentration in arterial blood, has a rather high between-subjects variability (range 0.24–0.60). This finding is in line with results obtained by Chen et al.^27^ and emphasizes the importance of an appropriate individualized partial volume correction to obtain an accurate and reproducible IDIF extraction. We also considered to optimize the correction for the left and right carotid artery independently, but this approach did not improve the partial volume correction compared to merging the left and right carotid artery VOIs.

To quantify [^18^F]JNJ-739 volume distributions in the human brain, both a two-tissue compartmental model and a Logan graphical analysis were validated previously^16^. When using an IDIF, a Logan graphical analysis is the preferred approach since it uses the time varying cumulated activity concentration in arterial blood rather than time dependent activity concentration as such. Therefore a Logan graphical analysis is assumed to be less dependent on the accurate determination of the rapidly changing peak of the input function which could be poorly estimated^23^. This is confirmed by several previous studies^25,38,39^. However, since metabolization of [^18^F]JNJ-739 is rather fast (only 30% ± 5% of parent tracer at 20 min post-injection), the contribution of later time points of the metabolite-corrected arterial plasma input function is limited such that cumulated activity concentrations at later time points, which are used for the Logan graphical analysis estimation, are more dependent on the accurate estimation of peak activity concentrations, shortly after tracer injection. Therefore, our approach is expected to be at least equally performant for tracers with a slower metabolization rate.

To avoid arterial cannulation, venous blood samples could be considered instead of arterial blood samples, not only to properly correct the IDIF for partial volume effects but also to accurately estimate the tracer metabolization rate and plasma to blood ratio^27^. Since we did not have venous blood data available for the current study using [^18^F]JNJ-739, we could not consider this approach as it needs a thorough validation. However, this approach can definitely be of value for PET tracers with similar venous and arterial metabolite fractions.

## Conclusions

In conclusion, a minimally invasive approach was studied to quantify [^18^F]JNJ-739 PET in the context of a dose occupancy and test–retest study by introducing an IDIF combining dynamic PET data with MR angiography acquired simultaneously on an integrated PET-MR system. Postdose V_T_ values and corresponding receptor occupancies, as well as test–retest repeatability of V_T_ were closely in line with values based on an invasive AIF approach, therefore allowing a reduction of invasive arterial sampling for repetitive quantitative PET imaging.

## Supplementary Information

Supplementary Information.

## Data Availability

The datasets generated during and/or analysed during the current study are not publicly available due to them containing information that could compromise research participant privacy/consent but are available from the corresponding author on reasonable request.
