# Supplementary material for: Minimally invasive quantification of cerebral P2X7R occupancy using dynamic [18F]JNJ-64413739 PET and MRA-driven image derived input function
Source: Sci Rep. 2021 Aug 9;11:16172. doi: 10.1038/s41598-021-95715-y (PMC8352986; doi:10.1038/s41598-021-95715-y)

Electronic Supplementary Material

Minimally invasive quantification of cerebral P2X7R occupancy using dynamic [^{18}F]JNJ-64413739 PET and MRA-driven image derived input function

Nathalie Mertens[1], Mark E. Schmidt[2], Anja Hijzen[2], Donatienne Van Weehaeghe[1], Paulien Ravenstijn[2], Marleen Depre[3], Jan de Hoon[3], Koen Van Laere[1], Michel Koole[1]

1 Nuclear Medicine and Molecular Imaging, Department of Imaging and Pathology, University Hospital and KU Leuven, Leuven, Belgium

2 Janssen Research & Development, Beerse, Belgium

3 Center for Clinical Pharmacology, University Hospital and KU Leuven, Leuven, Belgium

Supplementary Materials and Methods

Blood and PET data processing

During the dynamic PET acquisition, 22 arterial blood samples were manually obtained over a 90-minute period post-injection (10, 20, 30 40, 50, 60, 70, 80, 90, 100, 110, 120, 150, 180, 240, 300, 600, 1200, 1800, 2400, 3600 and 4800 seconds) to determine activity levels in whole blood and plasma. In parallel, 6 arterial blood samples obtained over a 90-minute period post-injection (2, 5, 15, 30, 60 and 80 minutes) were used to determine the percentage of intact tracer in plasma.

Arterial whole blood samples were centrifuged (5 min at 3000 rpm) to separate the plasma from the cellular fraction. Both the blood and plasma fractions were weighted and counted in an automated gamma counter. To obtain radioactivity values, the gamma counter measurements were scaled with a calibration factor obtained by simultaneously measuring a ^{68}Ge-sample with known activity. Next, the measured total radioactivity of each sample was divided by the corresponding weight and corrected for physical decay due to the time difference between the start of the PET scan and the start of the gamma counter measurements. This way, blood and plasma activity concentrations were obtained and used to determine the plasma to blood ratio.

In parallel, additional arterial blood samples were used for metabolite analysis, and centrifuged for 10 min at 3000 rmp to separate the plasma from the cellular fraction. Plasma samples were spiked with authentic JNJ-64413739 and the fraction of intact tracer and metabolites was quantified by RP-HPLC (Reversed-Phase High Performance Liquid Chromatography) while using an in-line UV detector combined with a NaI(TI) scintillation detector. Two fractions were obtained after elution, representing the fraction of polar metabolites and the fraction of the intact [^{18}F]JNJ-64413739 respectively. Radioactivity of both fractions was measured using an automated gamma counter as described previously to obtain the relative percentage of intact tracer.

The parent arterial plasma input function was obtained by applying plasma to blood ratio to the arterial whole blood input function and weighting the resulting input function with fraction of intact tracer. Regional time activity curves (TACs) were extracted by projecting a

simplified Hammers atlas on the dynamic PET data. For this purpose, PET data were spatially normalized to Montreal Neurological Institute (MNI) space using SPM8 and the 3D T1 MR. Volume of interests (VOIs) were restricted to gray matter by applying a simple threshold of 0.3 to the individual gray matter probability maps. This way, VOIs for the frontal, temporal, parietal and occipital cortex as well as the insula, anterior and posterior cingulate, striatum, thalamus, pallidum, hippocampus, amygdala, cerebellum, and brainstem were determined. From all cortical VOIs, a further composite cortical VOI was created to extract overall cortical TACs from the dynamic PET data.

PET-MR imaging and quantification

Dynamic PET images were corrected for motion using the PMOD (v4.1) software. For image registration, a Gaussian smoothing of 6 mm was applied, while a Powell minimization (tolerance 1.0E-4) and normalized mutual information as cost function with a sample rate of 2 voxels was used to align all frames of the dynamic PET scan with a reference frame. The reference frame was obtained by averaging the first 17 frames representing the first 5 minutes of the PET acquisition. Reslicing was performed using trilinear interpolation.

MRA and PET images were registered by performing a rigid co-registration using the PMOD (v4.1) software. As cost function, a normalized mutual information with Otsu thresholding of intensity values and a sample rate of 2 mm was used to estimate the 2D histogram. Additionally, a Powell minimization (tolerance 1.0E-4) was applied to align the two datasets. Finally, reslicing was done using trilinear interpolation.

Regional V_T values were estimated by using a Logan graphical analysis, determined as

$$\frac{\int_0^t C_T(\tau)d\tau}{C_T(t)} = V_T \frac{\int_0^t C_p(\tau)d\tau}{C_T(t)} + b$$

$C_T(t)$ represents the measured time activity curve of the tissue, while Cp(t) represents the plasma time activity curve. At equilibrium (t* = 48.5 min), regional V_T values were estimated by the slope of the Logan plot.

Supplementary Figures

Figure S1

A crisp figure explaining the study protocol. The dose occupancy study consists of three [18F]-739 PET-MR scan sessions: one baseline scan and two post dose scans acquired 4 hours after dosing (T_{max}). The test-retest study consists of two PET-MR scan sessions, acquired at least one week apart.

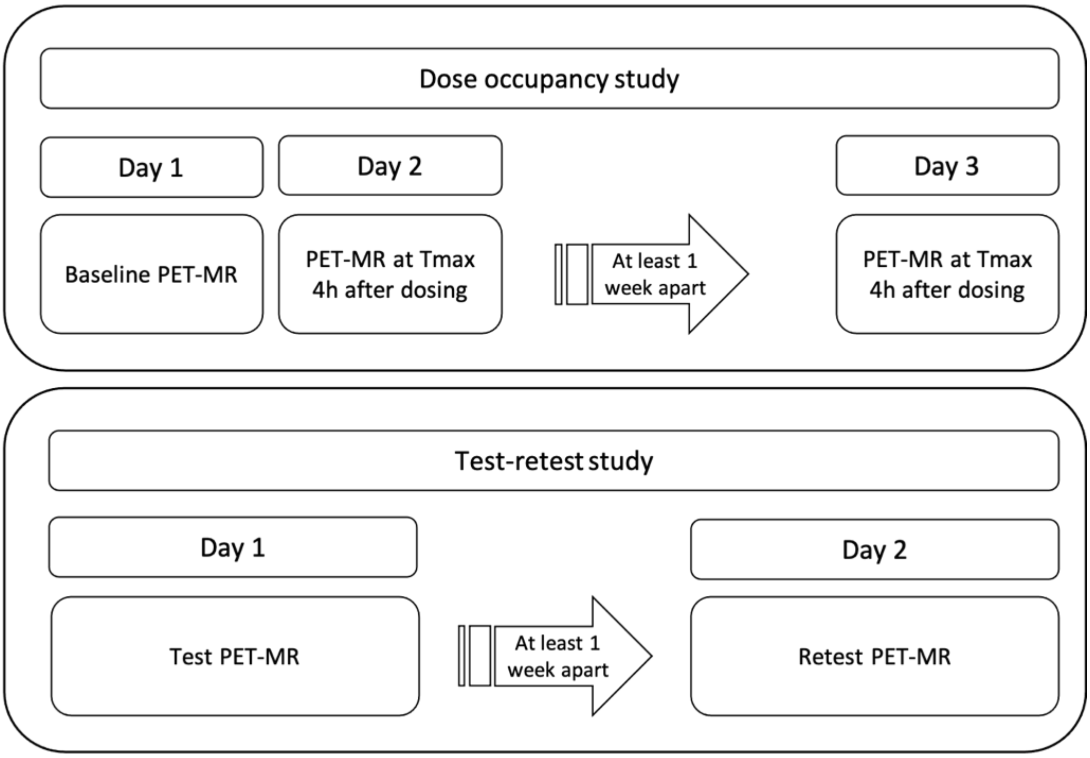

Figure S2

Tracer metabolite fraction of all subjects in the context of a dose occupancy study (n=6), showing very similar metabolite fractions under baseline and blocking conditions.

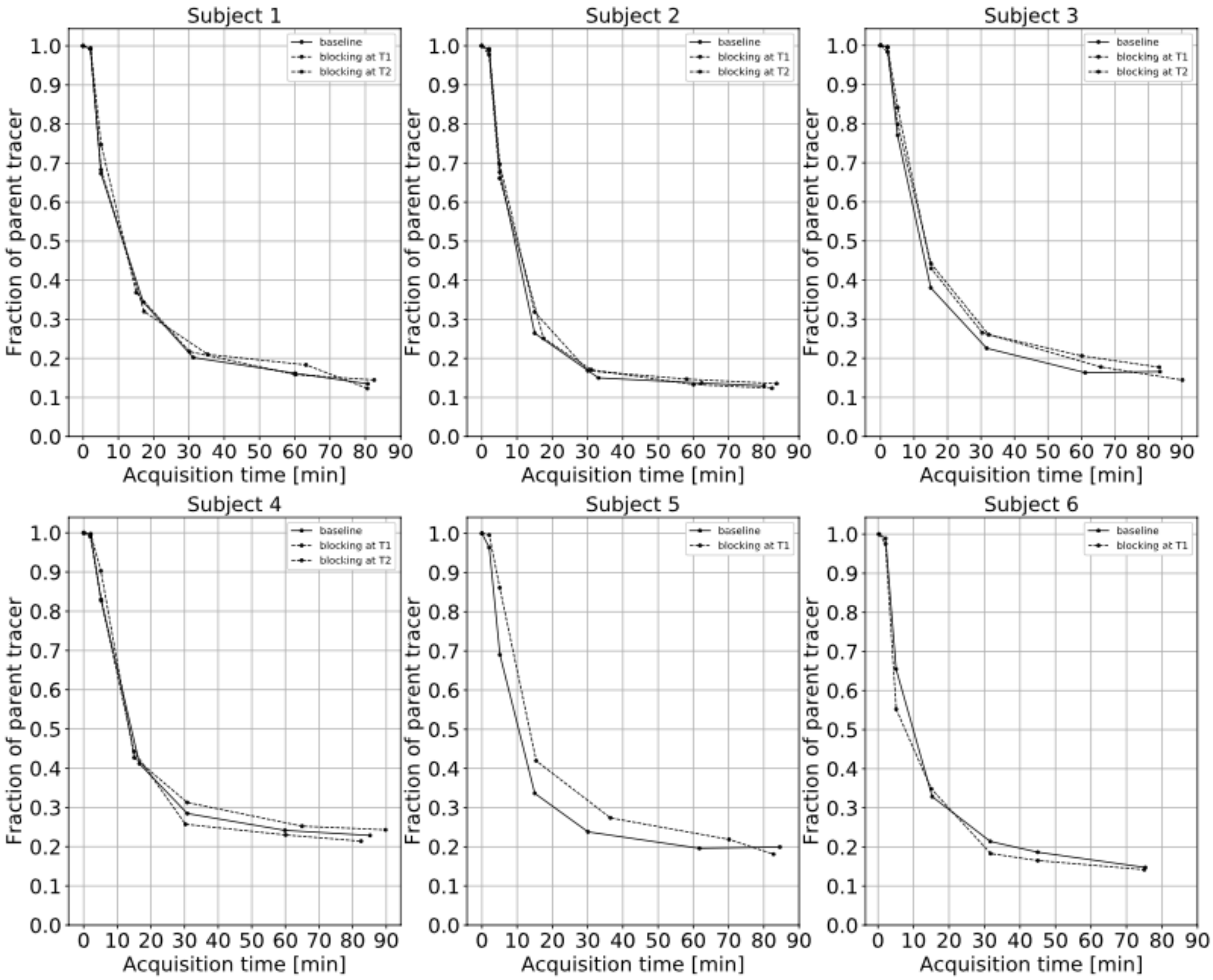

Figure S3

Baseline fractions of the intact tracer (left) and baseline blood to plasma ratio (right) for all subjects (n=10), showing high inter-subject variability.

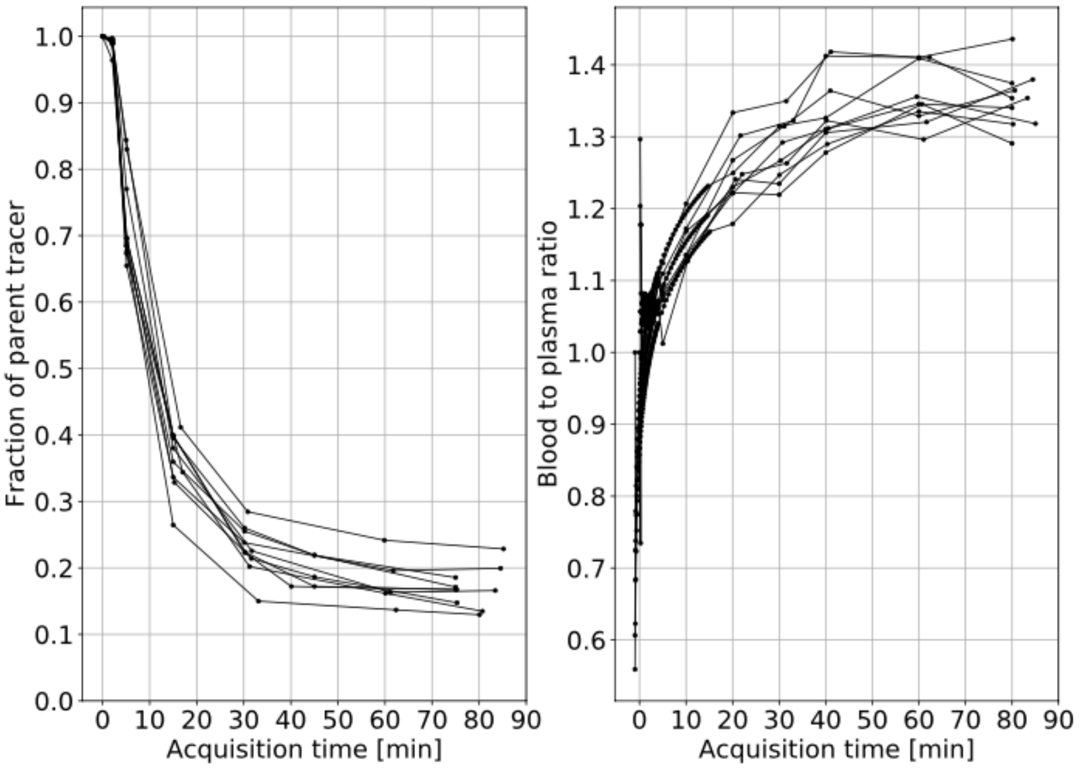

Supplement: Supplementary file 1 — Supplementary Information. [file 41598_2021_95715_MOESM1_ESM.pdf]
